# Study protocol: developing and evaluating an interactive web platform to teach children hunting, shooting and firearms safety: a randomized controlled trial

**DOI:** 10.1186/s12889-021-10345-3

**Published:** 2021-02-06

**Authors:** David C. Schwebel, D. Leann Long, Marissa Gowey, Joan Severson, Yefei He, Katelyn Trullinger

**Affiliations:** 1 Department of Psychology, University of Alabama at Birmingham, 1300 University Blvd, CH 415, Birmingham, AL 35294 USA; 2 Department of Biostatistics, University of Alabama at Birmingham, 1300 University Blvd, CH 415, Birmingham, USA; 3 Department of Pediatrics, University of Alabama at Birmingham, 1300 University Blvd, CH 415, Birmingham, AL 35294 USA; 4 Digital Artefacts, Iowa City, IA USA

**Keywords:** Firearms safety, Injury prevention, Child safety, Hunting and shooting, Clinical trial

## Abstract

**Background:**

Firearms injuries present a major pediatric public health challenge in the United States. This study protocol describes research to develop and then conduct a randomized clinical trial to evaluate ShootSafe, an interactive, engaging, educational website to teach children firearms safety.

ShootSafe has three primary goals: (a) teach children basic knowledge and skills needed to hunt, shoot, and use firearms safely; (b) help children learn and hone critical cognitive skills of impulse control and hypothetical thinking needed to use firearms safely; and (c) alter children’s perceptions about their own vulnerability and susceptibility to firearms-related injuries, the severity of those injuries, and their perceived norms about peer behavior surrounding firearms use. ShootSafe will accomplish these goals through a combination of interactive games plus short, impactful testimonial videos and short expert-led educational videos.

**Methods:**

Following website development, ShootSafe will be evaluated through a randomized controlled trial with 162 children ages 10–12, randomly assigning children to engage in ShootSafe or an active control website. Multiple self-report, computer-based, and behavioral measures will assess functioning at baseline, immediately following training, and at 4-month follow-up. Four sets of outcomes will be considered: firearms safety knowledge; cognitive skills in impulse control and hypothetical thinking; perceptions about firearms safety; and simulated behavior when handling, storing and transporting firearms. Training in both conditions will comprise two 45-min sessions.

**Discussion:**

If results are as hypothesized, ShootSafe offers potential as a theory-based program to teach children firearms safety in an accessible, engaging and educational manner. Translation into practice is highly feasible.

**Trial registration:**

The study protocol was registered on 11/10/20 at clinicaltrials.gov (NCT04622943).

## Background

Firearms injuries are a significant pediatric public health challenge in the United States. The Centers for Disease Control (CDC) estimates 803 children ages 0–15 were killed by firearms in the United States in 2018, and an additional 2422 children visited emergency rooms for treatment after a firearms injury [1]. Roughly half of children who are hospitalized for a firearm-related injury leave the hospital with a disability, creating long-term health, medical system, financial, family, and societal burden [2].

About one-third of firearms injuries to children under age 15 are due to unintentional causes rather than suicide or homicide [3]. Those injuries tally over 80 child deaths and 1200 serious injuries every year in the United States [4] and represent the current focus.

### Children and firearms

American children are routinely exposed to firearms in their homes. A study of 314 parent-child dyads seeking treatment at a rural health clinic in Alabama found that 201 of the homes (64%) had firearms present in their home [5]. Among those 201, 73% of children ages 5–10 and 79% of children ages 10–14 knew where the guns were stored. An equal number of children – 36% – in both age groups reported they had handled the firearms, including 52% of boys. Our laboratory’s research with 1561 fifth-graders in the Birmingham metropolitan area found that 31% of families reported having firearms in the home, with higher rates among families with Non-Hispanic White children than those with African-American or Hispanic children [6]. Just under half of the 440 families in that study with firearms in their homes (47%) stated they used the firearms for hunting.

Recognizing the methodological limitations of self-report data concerning firearms ownership and usage, a different study by our research team used home inspections to evaluate risk to Birmingham children [7]. In a study of 42 Birmingham-area families who agreed to have their homes inspected for broad child safety risks (mean child age = 15 years), 38% had a firearm present and 29% had a firearm present and unlocked. Among the 23% of families with rifles present, 79% stored the rifle unlocked, 61% stored ammunition in the same place as the rifle, and 15% stored the rifle unlocked and loaded with ammunition.

Online forums suggest children as young as age 5 and 6 sometimes start hunting and shooting with their parents. Most United States state laws permit children of any age to hunt legally under supervision by an adult. In many states, children can legally hunt unsupervised by an adult at any age if they complete a hunter education training program; the remaining states usually limit unsupervised hunting to children over age 10 or 12 years. Anecdotal reports widely cite the fact that children typically begin shooting and hunting during the elementary school years and often hunt and shoot alone or with peers and siblings by age 10 or 12, whether legal in their state or not.

### Firearms injuries while hunting and shooting

Among adults, between 25 and 33% of unintentional firearms injuries occur while hunting [8, 9]. In one case series of serious hunting injuries over a 2-year period, 10 of the 68 (15%) shooters were under age 15 and an additional 12 shooters (18%) were between the ages of 15–17 [8]. Half of the 22 children who unintentionally shot a person while hunting were being supervised by an adult while hunting, and half were not. 28% of the cases in the series were fatal.

Data on injuries and fatalities related to recreational shooting are less readily available, but one report suggests about 3% of unintentional firearms fatalities from 2003 to 2006 in selected US states were known to be the direct result of an incident during target shooting activities [10]. An additional 21% occurred during hunting, 11% while cleaning or loading the firearm, and 5% while carrying or handling it. A large portion (15%) were due to other or unknown causes and may also have been associated with hunting or shooting.

In fact, many unintentional firearms injuries are not directly related to hunting and shooting but are indirectly related because they occur immediately before or after hunting and shooting outings while firearms are being stored, transported, or cleaned. The largest portion of unintentional firearms fatalities reported in the Hemenway and colleagues paper [10] was due to playing with firearms (39% of deaths), and in over 70% of the fatal incidents, both the shooter and the victim were under the age of 25.

### The culture of hunting

Hunting culture has a long and proud history. Early Americans hunted for sustenance. Contemporary Americans still hunt to obtain food, but also for comradery, relaxation, an escape to nature, and sport. Most hunters prioritize safety in all they do, meticulously handling their firearms and cautiously shooting only when it is safe to do so. A small minority take dangerous risks, some of which lead to tragedy. Broad developmental research suggests children and teens may be more likely to take those risks [11], and that such risk taking can be thwarted with appropriate intervention [12].

RAND research suggests over 13 million Americans currently hunt with firearms [13]. One hallmark of the hunting culture is a desire to pass the joys of hunting to one’s children and grandchildren. The classic stereotype of a father taking his son out to shoot the boy’s first buck rings true in many American families, and offers not just nostalgia but also positive development of father-child relationships. Increasingly today, girls and women also are involved: Hunting reflects a family event [14–16].

Despite these positive features of hunting, the American hunting culture and tradition also creates danger when children are engaged. We review some risks below.

- The early start. Even casual hunters recognize their prey is often most active early in the morning, around dawn. Unfortunately, contemporary human society has evolved such that typical American children retire for sleep well past evening’s dusk. An early-morning awakening to hunt, therefore, is likely to cut children’s sleep time greatly short of the American Academy of Pediatrics recommendations for children ages 6–12 to sleep 9–12 h per night. Acute sleep restriction created by an early morning awakening is documented to have negative consequences on attention, impulse control, decision-making, and other neuro-cognitive functioning critical to maintaining safety around firearms [17–19].
- Once awakened and transported to the field, hunters often face adverse weather conditions. Deer hunting season throughout the United States falls in late autumn and winter. In most states, small game and fowl seasons run from late fall to early spring. Frigid temperatures, cold rain, and snow are commonplace. Due to physiological differences, children are less able than adults to regulate their body temperature to cold weather and are therefore more susceptible to negative health consequences from cold exposure [20]. Children may also resist wearing warm-weather gear, and may react emotionally and cognitively to feeling cold.
- Upon arriving in the field, there are various ways to hunt. One common strategy is hunting from a tree stand: the hunters are elevated and stable, waiting for the game to approach. This type of hunting requires extensive patience, as one sits and waits for the opportunity to shoot. For children – who have still-developing skills in patience and impulse control – it is also an invitation for error, as children may be excited when they sense movement in the forest and impulsively pull the gun’s trigger before ensuring they are aiming at an animal rather than another human. Other types of hunting are more active – they may involve walking through a forest or along a creek bed, for example – but still require patience, thoughtfulness and impulse control to maintain safety.
- Some adult hunters associate hunting with drinking alcohol. Children are less likely to imbibe, but they may be supervised by adults who are intoxicated, creating a situation where children absorb increased responsibility to behave safely because adult supervision is compromised. Child injury risk is elevated when supervising parents are intoxicated [21].
- Upon return home after a hunt, the full party (children included) is likely to be tired, cold, and hungry. This creates risk for safety sloppiness: will guns be transported and stored safety upon return? Will dangerous shortcuts be enacted? Will children be entrusted with adult tasks? Will younger siblings in the home, excited to see the rest of the family, encounter firearms?

### The culture of shooting

The culture of shooting evolved out of historic hunting practices. Today, many youth literally grow up with small arms like BB guns in their hands, which they use to shoot trees, signposts and backyard birds or rodents. As youth grow into their pre-teen years, shooting culture frequently evolves to more involved outings in the woods, at indoor or outdoor shooting ranges, or in organized competitions. Statista estimates over 30 million Americans engage in shooting firearms for sport [22].

Unlike hunting, shooting is common year-round and at all hours of the day. Reliable data sources are lacking, but injury rates appear to be lower also. Accurate reports of child injuries from nonpowder guns (e.g., BB, pellet, air guns) are available and indicate over 14,000 annual injuries requiring medical treatment, almost all of them unintentional and involving children, and many of them serious head and eye injuries [23–25]. Several of the risks present while hunting with children – adverse weather conditions, need for patience and impulse control, adult alcohol use, and sloppy safety upon return home, for example – may emerge during and after shooting excursions just as they do with hunting.

### Previous research: existing firearms safety programs for children

A large portion of existing child firearms safety programs focus on changing adult behavior through strategies such as safe firearms storage rather than on changing children’s behavior with firearms. Two recent systematic reviews, which identified 12 and 10 studies respectively targeting children (and 9 others targeting adolescents in the Ngo et al. review), suggest most existing programs to teach children firearms safety are ineffective [26, 27]. A surprising number of published empirical trials yield either null results or results that document capacity to teach children basic knowledge but not translate that knowledge into safer behavior.

Reviewed studies also were critiqued as suffering from several weaknesses, including absence of theoretical basis to achieve behavior change and significant methodological concerns like small sample sizes, lack of rigorous research designs to evaluate the interventions (e.g., no control groups), and a focus on knowledge-based outcomes without adequate measurement of behavioral outcomes [26, 27]. None of the reviewed studies used technology-based intervention programs that offer the engaging, interactive, and experiential training medium today’s children prefer. (The Eddie Eagle program, developed by the National Rifle Association, has recently been transferred from its original classroom-based format to a website training program for children about ages 4–9 [28], but the efficacy of that website as a training tool has not been evaluated in a published clinical trial).

Despite the discouraging results of previous research, a smattering of studies offer promising results to guide the current study. Perhaps most influential is a series of studies by Miltenberger and colleagues that test the use of active learning strategies, generally delivered in small-group or classroom settings, to teach children firearms safety [29–36]. In all cases, these studies use small sample sizes (largest *N* = 45 and many *N*s < 10). The findings generally suggest children exposed to theory-based active-learning activities involving behavioral strategies like modeling, rehearsal and feedback successfully learn both relevant firearms safety skills as well as displaying appropriate behavior in role-play scenarios following the training. We adopt these active-learning behavioral strategies into an internet-based delivery system for ShootSafe.

### Present research

Based on existing knowledge and research, we will develop and then evaluate ShootSafe, an innovative and interactive website that engages children to learn firearms safety in an experiential, educational and enjoyable manner. We have three primary educational goals: (1) teach children the basic skills they need to hunt, shoot, and use firearms in a safe manner; (2) help children learn and hone critical cognitive skills of impulse control and hypothetical thinking they need to use, store, transport, clean and handle firearms safely; and (3) alter children’s perceptions about their own vulnerability and susceptibility to firearms-related injuries, the potential severity of those injuries, and their perceived norms about peer behavior surrounding firearms use. Theory-driven games, activities, videos and other features on ShootSafe will be developed to achieve those goals.

ShootSafe will primarily target children ages 10 to 12. This is an age group that begins to hunt and shoot independent of adult supervision, as well as a developmental stage when children are actively learning and developing two cognitive skills critical to safety with firearms: (a) executive function (especially impulse control) and (b) hypothetical thinking (especially anticipation of future events, deducing possible occurrences, and planning). In classical child development theory, Piaget referred to these developments as entry into the formal operations stage, a time when children develop cognitive skills like logical thought, deductive reasoning, and executive function [37]. In contemporary theory, neuroscientists suggest the pre-adolescent years involve a surge in production of gray matter in the brain’s frontal lobe, production that is linked to rapidly-developing skills in executive functions like planning, organizing, impulse control, problem-solving, and deductive reasoning [38]. Thus, ShootSafe capitalizes on natural child development to accelerate cognitive skill development in domains relevant to safe use, transport and storage of firearms. In Vygoskian terms, we use technology to scaffold development of critical cognitive skills within a targeted developmental zone of proximal development [39].

## Methods/design

### ShootSafe website

The ShootSafe website, currently in development, is designed to accomplish three primary goals: (1) transmit knowledge about hunting and shooting safety, (2) hone relevant cognitive skills to safely hunt, shoot, handle and store firearms, and [3] revise perceptions about firearms safety (See Table 1).

**Table 1:** ShootSafe Website Components

ShootSafe goals are to:	
1. Transmit knowledge [via expert videos, trivia games]	
a. Safe preparation	
b. Safe transport	
c. Safety in the field	
d. Safe storage	
2. Hone relevant cognitive skills [via interactive games]	
a. Executive function – impulse control, patience	
b. Hypothetical thinking – anticipate future, deduce what could happen, plan ahead	
3. Alter perceptions about firearms safety [via expert videos, peer testimonials]	
a. Increase self-efficacy	
b. Increase perceived vulnerability and susceptibility	
c. Recognize severity of errors	
d. Change perception of peer normative behavior	

#### Transmit knowledge

The first step to any safety intervention is ensuring the individual has requisite knowledge to engage in safe behavior. Safe firearms use requires substantial knowledge about guidelines, rules, and recommendations. ShootSafe will convey these lessons repeatedly to reinforce learning, and through multiple mechanisms to reach youth. Primary modes of delivery are through “expert videos”, in which expert hunters and shooters offer their advice and suggestions, and through trivia games that offer an engaging, rewarding and entertaining means of transmitting knowledge to youth.

#### Hone relevant cognitive skills

Leveraging the cognitive growth that occurs during the target age range, ShootSafe will incorporate games that have the explicit purpose of honing two key sets of cognitive skills required for proper use of firearms: executive function (with specific focus on impulse control and patience) and hypothetical thinking (with focus on anticipating possible future outcomes, deducing risky events that could possibly occur, and planning ahead).

Executive function skills like impulse control and patience actively develop and mature during the pre-teen years [38, 40, 41]. They are highly relevant to firearms safety, as impulsive or impatient action can lead to tragic results. The skills also are amenable to improvement through training [42, 43].

Like impulse control and patience, hypothetical thinking actively develops and matures during the pre-teen years [44] and has direct relevance to firearms safety. Labeled variously in different literatures, we are interested in a construct that incorporates consequentiality (what happens as a consequence of a decision?), fluency and flexibility (are children creative and flexible to deduce alternative and unique outcomes of a situation?), and divergent thinking (can children think in alternative ways?). Poor anticipation of where a bullet might travel if the target is missed can lead to disastrous mistakes when, for example, a roadway with moving vehicles lies behind the target. Similarly, poor anticipation of the fact that a younger sibling might discover an unsecured firearm that is left in the front hallway upon return from a hunting trip can lead to firearms injury or death to the sibling or others. Training to help children learn hypothetical thinking, planning, and deducing possible negative outcomes has proven successful in classroom settings [45, 46] as well as through computer games [47].

#### Revise perceptions about firearms safety

Adolescents are commonly reported to take more risks than adults, including risks involving broad personal health and safety [12, 48] and those involving firearms injury specifically [49]. To accomplish desired changes in perceptions about safety, ShootSafe will work to alter youth perceptions surrounding four health behavior change theory topics: (1) increased self-efficacy to behave safely, (2) increased perceptions of vulnerability and susceptibility to injury events, (3) increased recognition of the severity of an error, and (4) changed perception of norms about how peers behave.

Increasing self-efficacy to change health-related behavior forms a prominent piece of several health behavior change theories, including the Health Belief Model [50], the theory of planned behavior (which refers to the concept as perceived behavioral control) [51, 52], and Bandura’s social cognition theory [53, 54]. In all cases, individuals need to perceive they have both the knowledge and the capacity to make health-related behavior change. ShootSafe will transmit relevant knowledge through the website features, inspiring increased self-efficacy through content-relevant games that are challenging yet accomplishable.

Models of health behavior change also almost universally cite the need for individuals to perceive the fact that they are vulnerable or susceptible to negative health outcomes if they are to change their behavior. Such arguments appear prominently, for example, in Weinstein’s unrealistic optimism theory [55, 56] and in the Health Belief Model [50]. A powerful strategy to alter perceived vulnerability or susceptibility to injury is through the use of testimonials [57]. ShootSafe therefore includes videos delivered by child actors who share stories of “firearms accidents they have experienced”, providing realistic, emotional and relatable stories about the risks of unsafe firearms use, transport, handling and storage, plus actions that could be taken to reduce risk.

Also relevant to how individual perceptions influence health behavior is the individual’s perception of the severity of an error [50, 58]. Would a mistake lead to a minor mishap or to a fatal injury? Convincing youth that a simple mistake can lead not just to a cut or scrape but to death for oneself or someone else is a powerful message that must be delivered repeatedly, gravely, and emotionally. Given the immature cognitive skill of a pre-teen to anticipate the future and think hypothetically, conveyance and deep recognition of the potential gravity and severity of the consequence of a mistake must be delivered. ShootSafe will transmit these messages through both expert and peer testimonial videos.

Finally, health behavior change theory stresses the need for perceptions that safe behavior is normative. Most prominent in Ajzen’s Theory of Planned Behavior [51, 52], an interventionist must convince youth that safe behaviors are typical and normal among their peers, and that they would be an outlier if they did not engage in those safe behaviors also. ShootSafe’s testimonial videos will deliver this message repeatedly, and those messages will be supplemented by expert videos and by role-playing games involving youth engaged in firearms-related activities.

#### ShootSafe components

Substantial research indicates that children play, enjoy, and learn from educational games and activities [42, 59–61]. ShootSafe will offer a gamified environment including games and videos that engage children, allow them to earn points and achieve new levels, and offer sufficient challenge so they want to return to re-engage.

Games will include trivia games to encourage and enable basic learning of facts, games that require practice of the challenging task of controlling impulses to respond to appetitive stimuli, and games that require practice of hypothetical thinking and anticipation of possible future events. Some games will involve hunting and shooting themes and activities, but many will reflect other engaging activities that teach the desired and relevant skills.

ShootSafe’s videos will be divided into two categories, testimonials from peers and expert videos. Testimonials will be presented by child actors who deliver diverse and realistic situations and stories, all emotional, about real-life firearms-related injuries and deaths. Expert videos will be delivered by firearms safety experts who share their expertise in the four focus domains of safe preparation, safe transport, safety in the field, and safe storage.

ShootSafe will also incorporate two other unique features. First, a reward system will provide points to children that motivate them to perform well on the website. Second, the point system will drive tailored messaging to parents. When children earn points or achieve new levels, a message will be sent to their parents, encouraging parents to congratulate children and reinforce targeted information about children’s lessons. We view this messaging as a strategy to keep today’s overextended parents informed about their children’s learning but not inundate them with lengthy material they do not read or process.

### Randomized design study

**Fig. 1:**
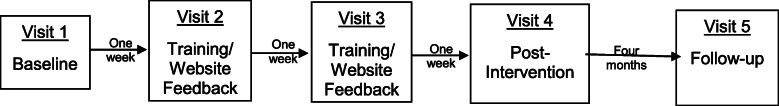
Timeline of Study Visits

After the website is fully developed, we will conduct a repeated measures randomized control design experiment with active intervention and control groups assessed during pre-test (baseline), post-test, and 4-month follow-up laboratory visits. The protocol is registered at clinicaltrials.gov (NCT04622943). As detailed below, children will be recruited from the Birmingham, Alabama area to participate. Following a baseline assessment, children will be randomly assigned to use either the intervention ShootSafe website or a control nutrition website (nourishinteractive.com) during two training visit appointments, each 45 min long. A 1:1 allocation ratio will be implemented with randomization achieved by the research team through a random number generator and randomized order concealed to all parties prior to assignment during the first training visit. To measure learning and retention of lessons and perceptions, children will return to the laboratory for a post-intervention visit and then a follow-up visit four months later. Participant engagement is displayed graphically in Fig. 1, and details of recruitment and retention strategies, plus all assessment measures and the data analysis plan, appear below.

#### Participants

One hundred sixty-two participants ages 10–12 will participate. We will recruit through local advertising and anticipate representative local diversity among those exposed to firearms in terms of race, ethnicity, family Socioeconomic Status and rurality. Eligible families will be those with English fluency, children falling in the appropriate age group, and children who do not have disabilities prohibiting valid study participation (e.g., blindness, intellectual disability). We also will exclude children who have not been exposed to firearms through personal experience with firearms in the home or by engagement in hunting or shooting activities. Given the demographics of eligible families both nationally and in the local area [62], we anticipate a modestly racially diverse (~ 30% ethnic minority), gender-balanced sample. Participants will provide informed consent (parents)/assent (children) and will be compensated for their time.

#### General protocol

Following recruitment, families will visit the laboratory at a convenient time, complete consenting with a research assistant, and then engage in a baseline battery with a trained research assistant to assess knowledge, perceptions and behavior relevant to firearms safety. At the end of the visit, families will be randomly assigned to either the intervention ShootSafe group or the control nutrition group. Training will occur across two monitored 45-min laboratory sessions over a two-week period. At that point, families will return to the laboratory to complete a post-intervention battery similar to the baseline assessment. Finally, families will return four months later for a follow-up assessment appointment.

The protocol for the baseline, post-intervention, and follow-up visits will run similarly. Children will complete a mix of self-report, computer-based, and behavioral tasks, all described below. The protocol for training visits will be straightforward. Upon arrival, children will be stationed at a desk and monitored through a one-way mirror as they engage in the website. If participants are witnessed to wander off-site or appear highly distracted (e.g., looking away from the program for lengthy periods of time), the experimenter will interrupt to re-direct the child back on task. Apparent attention and fidgeting will be rated by the experimenter. Substantial noncompliance with the intervention will result in trial dismissal.

#### Interventions: ShootSafe and Nourish Interactive

Given the novelty of ShootSafe and following typical practice in pharmaceutical and other early-phase health intervention clinical trials, we will conduct a trial that falls closer to the explanatory than pragmatic side of the explanatory-pragmatic clinical trial continuum [63]. All training will occur in a laboratory setting where we can monitor usage and control exposure to the intervention. Children in both intervention groups will use the websites during two 45-min laboratory training sessions. 

As detailed elsewhere, the active ShootSafe intervention will offer a wide range of games and videos for children to try, and we will allow children to self-direct to their preferred activities. The comparison group will visit nourishinteractive.com, a website that provides educational games and activities relevant to child nutrition and exercise. Of similar size/scope to ShootSafe, nourishinteractive.com offers extensive games, stories, and interactive activities for children in a range of age groups, including our target age. 

#### Measures

Website use and engagement will be assessed via children’s attention and fidgeting while engaging in the website using objective behavior rating scales developed previously in our laboratory [64, 65]. Further, children will complete surveys concerning their impressions and perceptions about the website they engaged within both immediately after web use during training visits and during the post-intervention visit.

Demographics will be assessed via parent questionnaire. Firearms use, training and experience will be assessed through both child- and parent-report, offering information on the child’s experience, habits and practices using firearms both alone and while supervised. We also will gather information about previous firearms safety training. Similarly, online game use and experience will be assessed through child- and parent-report.

To test whether intelligence or temperament influence efficacy of the websites, we will conduct brief screens of intelligence using WISC-V (Wechsler Intelligence Scales for Children – V) subtests [66] and child temperament through the parent- and child-report versions of the EATQ-R (Early Adolescent Temperament Questionnaire – Revised) [67, 68].

Perceptions about vulnerability, susceptibility, and potential severity of firearms injury, plus peer norms, will be surveyed via self-report survey from both children and parents. We also will assess parent and child perceptions of normative peer behavior about firearms use and storage. All perceptions about firearms safety will be assessed at baseline, post-intervention, and follow-up.

Children’s cognition will be assessed using multiple methods. Impulse control will be assessed both with neuropsychological and laboratory measures. A brief neuropsychological battery will be constructed using age-appropriate adapted Stroop and Go-No Go tasks. The Stroop task asks children to inhibit one cognitive inclination that emerges (e.g., read the word, “blue”, that is presented) while processing a second impeding set of stimuli (e.g., the word “blue” is presented in red writing) [69]. In that example, children would thus be requested to state the color of the writing (red), a task requiring inhibition of the desire to read the word (blue) instead of stating the color of its letters.

The Go-No Go task is designed primarily as a measure of inhibitory control. Computer-driven, it presents a series of stimuli, some of which require a response (the “go” stimuli) and others of which require inhibition of the response (the “no go” stimuli) [70]. The test yields several outcome measures, including correct responses to the “go” stimulus, omission errors (failure to respond to the “go” stimulus), commission errors (false alarms in responding to the “no-go” stimulus), correct rejections of the “no-go” stimulus, and reaction time. We will focus especially on commission errors and response time to the “go” stimuli, which are considered core measures of inhibitory control [71].

Behavioral measures of impulse control will comprise a 7-task behavioral battery developed previously through a mix of novel and adapted activities [72]. As shown in Table 2, tasks in the battery assess ability to slow fine (Draw-a-Circle) and gross (Walk-a-Line) motor movement, control verbal impulses (Long Speech), and delay gratification for short (Peeking) and long (Money, previously called Pencils) latencies. Other tasks assess speed of decision-making (Prize-Choosing) and behavioral surgency (Newspaper Ripping).

**Table 2:** Behavioral Battery of Impulsivity and Inhibitory Control

Task	Activity	Outcomes
Draw-a-Circle	Draw a circle on paper template, as slow as possible	Difference score, “regular” – “slow” drawing
Walk-a-Line	Walk along line marked on floor as slow as possible	Difference score, “regular” – “slow” walking
Long Speech	Listen to long and boring soliloquy from researcher	# interruptions; time to 1st interruption
Peeking	Wait in room while researcher leaves, with “treat” hidden under a cup	Does child “peek”? Does child eat treat? Latency to first peek
Money	Choose between receiving a single $1 bill immediately vs. two $1 bills at the next visit	Selection of immediate vs delayed reward
Prize-Choosing	Child chooses prizes out of a very large bin full of attractive age-appropriate prizes	Timed latency to prize selection
Newspaper Ripping	Child is given a section of newspaper and asked to rip it into small strips	Time spent; Coding of mood/exuberance; Number of strips created

Hypothetical thinking will also be assessed through a combination of neuropsychological and laboratory measures. Two neuropsychological measures will be used. First, we will administer the picture arrangement subtest from the WISC-III [73]. Administration of this measure involves showing children a series of illustrations in mixed-up order; children are asked to place the illustrations in the appropriate order to represent a logical flow of events. Successful engagement in the task requires key cognitive skills, therefore, of anticipating events that might flow from other events. The picture arrangement subtest was dropped from subsequent versions of the Wechsler intelligence scales to minimize the influences of motor coordination and rapid processing (time bonuses) on broader intelligence scores [74], but both motor coordination and rapid processing of information are relevant to safety with firearms. Further, the picture arrangement subtest of the WISC-III shows excellent validity with other measures of intelligence, but not with measures of social intelligence or social processing [73, 75].

The second neuropsychological measure will be an evaluation of creative and divergent thinking. We will use subtests of the Torrance Tests of Creative Thinking (TTCT) to assess these traits [76]. In particular, we are interested in children’s fluency and flexibility: are children creative and flexible in identifying alternative and unique solutions and outcomes to events and situations? These skills will be evaluated using the Making Guesses, Just Suppose, and Common Problems TTCT subtests. The laboratory measure of hypothetical thinking will be conducted via a dollhouse simulation task and is detailed below.

We will assess children’s knowledge using three techniques. Two strategies will be adapted from previous work: (a) a basic written “quiz” concerning firearms safety and (b) a series of photographs showing various safe and unsafe situations, in which children respond whether the situation is safe or not, offering a second, more visual assessment of their knowledge about safety [77–79]. We also will use the photographs as a crude measure of intended behavior surrounding firearms by asking children whether they would engage in that behavior or not.

The third measure of knowledge will also be used to assess both children’s knowledge and their intended behavior in various scenarios. Offering a richer assessment of behavior than intentions based on a photograph, we will develop a role-play simulation based loosely on our research simulating situations relevant to child dog bite risk through interaction with a live dog [78]. Specifically, we will arrange in our laboratory a replicated hunting and shooting scene, re-creating the scenery of a forest. Small game will be built around the structure of toy remote-controlled cars. Children will be given toy guns and asked to shoot both game and targets, replicating both hunting and shooting activities as part of the simulation. They also will return to a different room to “store” their firearms safely at the end of the activity. We will create situations with possible risk (e.g., shooting at a target with people in the background vs not) as well as recording proper transport of the weapon (e.g., muzzle pointing up or down) and proper storage (at end of task, is gun stored and locked in cabinet, separate from ammunition?). All behavior will be videotaped and subsequently coded using an objective coding scheme.

Finally, experimental laboratory measures to assess both hypothetical thinking and behavior while using firearms will be conducted using dollhouse simulations. In these scenarios, children will hear a narrative that is left unfinished and will be asked to complete the story, allowing us to evaluate children’s ability to think hypothetically. From a social development perspective, these tasks evaluate the cognitive skill of consequentiality – understanding what might happen as a consequence of a decision [80, 81]. How and when do children process, consider and understand the potential consequences of a decision? To conduct the assessment, we will present the short vignettes verbally following a structured script, and simultaneously act out the scenario using dollhouses, scenery, characters and other props from the Playmobil toy series (Zirndorf, Germany). Primary characters will be matched by gender and skin tone to the study participant. Replicating protocols from previous work using this methodology [77, 78], children’s responses to the scenarios will be video-recorded and subsequently scored using an objective coding scheme.

#### Patient and public involvement

A 5-person Advisory Board comprised of experts in firearms safety, education, child development, and instructional design were selected from the public to advise the research team on the development and evaluation of ShootSafe.

### Data management and statistical analysis

Data entry will be primarily electronic, and data will be stored on secure servers to preserve confidentiality. Data analysis will be conducted with condition masked. Descriptive analyses will be conducted first to examine the distributions of key variables and identify any unusual cases or outliers. Most outcome variables are expected to be normally distributed, but variables with substantially skewed distributions will be appropriately transformed so that linear models may be applied without violating assumptions.

Following inspection and interpretation of descriptive statistics, primary inferential data analyses will address the study’s specific aims. Primary analyses will be conducted with the full sample using an intention-to-treat analytic approach.

The first aim is to evaluate whether the website improves children’s knowledge about firearms safety. This aim will be measured through the scores from the quiz, photograph, and role play simulation tasks. We will standardize each outcome variable and evaluate through correlation matrices and factor analysis to determine if they appear to be measuring the same underlying construct. If so, they will be aggregated. Otherwise, they will be assessed as independent outcome variables.

Linear mixed models will evaluate our primary hypotheses. Linear mixed models were selected primarily because they offer the ability to allow for correlation within each child based on all three of the child’s measurements. Group differences will be tested by fitting the following model:

$$ \mathrm{E}\ \left[{\mathrm{Y}}_{\mathrm{ij}}|\ \mathrm{X},\mathrm{Z}\right]={\beta}_0+{\beta}_1{\mathrm{X}}_{\mathrm{group}}+{\beta}_2{\mathrm{X}}_{\mathrm{time}}+{\beta}_3{\mathrm{X}}_{\mathrm{group}}\ast {\mathrm{X}}_{\mathrm{time}} $$

where time will be entered into the model as a categorical variable (baseline vs post vs follow-up) utilizing effect cell coding so that we can determine whether changes in the outcomes between different time points differ by intervention group. Of greatest interest will be β_3_, as this parameter will tell us whether the effect of the intervention group differs between baseline, post-intervention and follow-up time points. If we find that β_3_ is significant, we will perform orthogonal contrasts to determine which specific time points differ from each other. To increase statistical efficiency, we will use previous literature to identify covariates that may be highly associated with the outcome before statistical analysis, and those covariates will be included in the linear model [82, 83].

The second aim is to evaluate efficacy of the program to improve relevant cognitive skills in two domains, impulse control and hypothetical thinking. As detailed above, each cognitive skill construct will be assessed via multiple measures so we will standardize and then aggregate multiple measures for analysis, conducting separate analyses for any measures that do not aggregate well. Then, we will fit the same linear mixed model for each outcome variable.

The third aim is to evaluate the program’s efficacy to improve children’s perceptions of vulnerability, susceptibility and severity of injury, plus their perception of normative peer behavior. Each construct will be assessed through self-report. The final aim, to evaluate the program’s efficacy to change children’s behavior around firearms, will be evaluated through the photograph, dollhouse simulation and role-play simulation tasks. These last two aims will be evaluated identically to the first two.

We anticipate missing data among the dataset for various reasons, including attrition from the study, rare participant refusal to conduct particular tests or answer particular questions and rare equipment failure or experimenter error. The planned linear mixed models allow the use of all observed data, including data from participants with incomplete missing outcome measurement at follow-up, with the relatively mild assumption of missing at random [84]. We will handle missing covariate data through multiple imputation or other appropriate analytic strategies.

#### Power

**Fig. 2:**
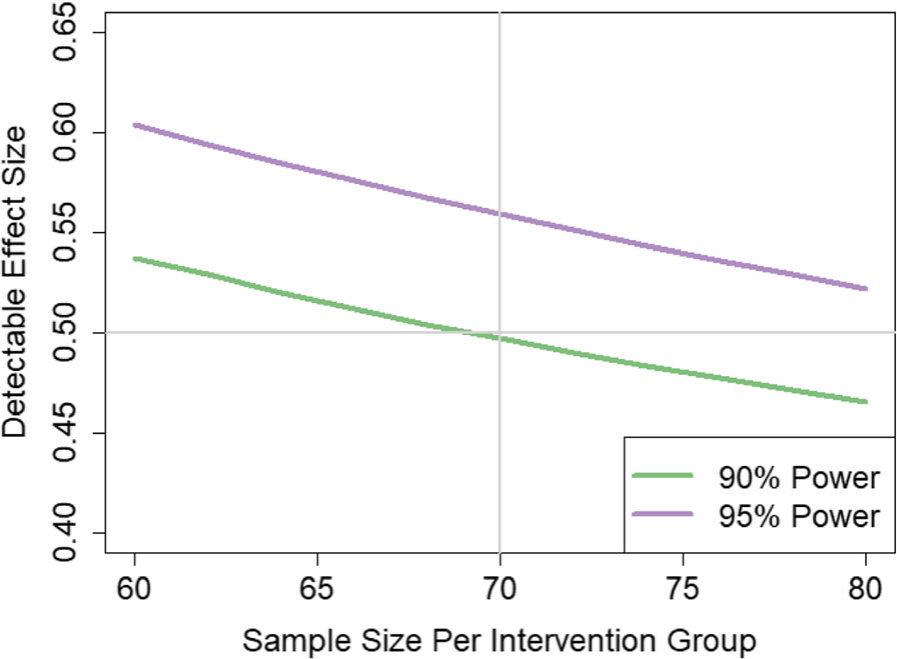
Detectable Effect Size by Sample Size at 90 and 95% Power

A power analysis was conducted to determine sample size using PASS 14 (Power Analysis and Sample Size) software [85]. Based on previous work [77, 78], we conservatively assumed change of 3 (SD = 2) in the ShootSafe website condition and 2 (SD = 2) in the comparison condition, yielding an anticipated medium effect size of 0.5. That is, we have power to detect a difference of 0.5 standard deviations between the ShootSafe website and control condition groups. Using 2 tails and α = .05 and assuming an independent samples t-test is conducted, a sample size of 70 per group provides 90% power to detect an effect size as small as 0.5 [86, 87]. With the same assumptions and retaining *n* = 70 per group, we estimate 95% power to detect an effect size of at least 0.56 (Fig. 2). As we are using a linear mixed model to assess the specific aims rather than a t-test, we will ensure that power is higher yet than that determined above since we will incorporate the observed correlation between the trials. Conservatively inflating 15% to account for attrition, therefore, our proposed sample size of 162 is amply large to test our hypotheses.

### Study and data monitoring

A data safety monitoring board will be established as an independent monitor of the study and of participant safety. The board will be comprised of three individuals independent from the study team and the sponsor, each with professional exertise relevant to the project. Interim analyses will be conducted at least annually and reviewed by the study statistician and the data safety monitoring board. All adverse events will be reported to the Institutional Review Board (IRB). If there is unanticipated evidence the intervention may be having adverse effects, the study protocol will be immediately suspended. Conversations will be held with the data safety monitoring board and other relevant parties, including the sponsor and the IRB, to determine whether the protocol is continued, amended or terminated, with participant safety of foremost concern.

Protocol amendments will be reported to all relevant parties.

### Dissemination

Results will be disseminated through peer-reviewed scientific reports prepared by the study team without use of professional writers and without influence from the study sponsor. We will work with our university media team to share results with the general public through the mass media, also. The study protocol and data will be made available to qualifed individuals upon request.

## Discussion

If the study hypotheses prove true, we view ShootSafe as a tool with potential for broad dissemination. The next logical step would be conducting a larger randomized trial, followed by partnership with appropriate government, industry, or non-profit groups to facilitate distribution for wide national and international use.

## Data Availability

The datasets that will be collected during the current study will be available from the corresponding author on reasonable request.

## Abbreviations

CDC: Centers for Disease Control and Prevention
HHS: United States Department of Health and Human Services
WISC: Wechsler Intelligence Scales for Children
EATQ-R: Early Adolescent Temperament Questionnaire – Revised
TTCT: Torrance Tests of Creative Thinking
PASS 14: Power Analysis and Sample Size
IRB: Institutional Review Board
